# Isolation, Genomic Characterization, and Immunogenicity Evaluation of a G9P[23] Porcine Rotavirus Strain

**DOI:** 10.3390/vetsci12020180

**Published:** 2025-02-18

**Authors:** Zixuan Wang, Wen Huang, Gengxuan Yan, Yuan Tian, Chune Wang, Xue Mao, Meng Sun, Lu Zhou, Chong Yu, Haihua Xia

**Affiliations:** 1 Institute of Microbiology, Heilongjiang Academy of Sciences, Harbin 150010, China; 2 National Key Laboratory of Agricultural Microbiology, College of Veterinary Medicine, Huazhong Agricultural University, Wuhan 430070, China; 3 National Institutes for Food and Drug Control, Beijing 100050, China

**Keywords:** porcine rotavirus, isolation, genome analysis, inactivated vaccine, neutralizing antibodies, cross-neutralization

## Abstract

(1) Background: Rotavirus (RV) is the primary pathogen causing diarrhea in infants and young children. The G9-type is an emerging genotype; however, its isolation is challenging, and its immunogenicity to piglets is poorly understood. This study aimed to isolate, characterize, and evaluate the immunogenicity of a PoRVA strain, RHeN2, obtained from the diarrhea samples of piglets from a pig farm in Henan Province, China. (2) Methods: Porcine rotavirus A (PoRVA) was isolated from diarrheal samples of piglets on a farm in Henan Province, China, through sequential passaging on MA-104 cells. Its immunogenicity and cross-neutralization potential were evaluated. Inactivated vaccines were prepared using two agents (formaldehyde and binary ethyleneimine, BEI) and administered intramuscularly to 28-day-old piglets. Dulbecco’s Modified Eagle Medium (DMEM) served as the control. (3) Results: A PoRVA strain, G9P[23] type, was successfully isolated and named RHeN2. Full-genome Sanger sequencing revealed the genetic constellation of this strain as G9-P[23]-I5-R1-C1- M1-A8-N1-T1-E1-H1. In addition, the titers of neutralizing antibodies peaked at 45 days post-vaccination, and the immunogenicity of the BEI-inactivated group of vaccines was better than that of the formaldehyde-inactivated group. The RHeN2 (G9P[23]) strain demonstrated a strong cross-neutralization ability against the same G9P[23] and G4P[23] strains and a stronger cross-neutralizing ability against the P[23] strains than against the P[7] strains. (4) Conclusion: The newly isolated G9P[23] strain demonstrates favorable immunogenicity, holding potential as a G9-type vaccine candidate.

## Simple Summary

Species A rotaviruses are major pathogens that cause diarrhea in young children and animals. Porcine rotavirus A (PoRVA) G9-type is an emerging genotype with the potential for cross-species transmission between humans and swine, which causes economic losses to the livestock industry and threatens human and animal health. This study isolates and characterizes a novel porcine rotavirus strain, RHeN2 (G9P[23]), and evaluates its immunogenicity using two inactivation methods. It demonstrates the potential of RHeN2 as a candidate for a G9-type porcine rotavirus vaccine and identifies binary ethyleneimine as a superior inactivator compared with formaldehyde for preserving immunogenicity. The findings provide insights for the selection of inactivating agents for PoRVA vaccines and the development of G9-type vaccines.

## 1. Introduction

Rotavirus (RV), a double-stranded RNA virus from the Reoviridae family, has a genome comprising 11 RNA segments encoding six structural (VP1–VP4, VP6, and VP7) and six nonstructural (NSP1–NSP5/6) proteins [1]. RV is classified into 10 species (RVA–RVJ) based on the VP6 antigenic profile, all of which infect animals [2]. Five species (A, B, C, E, and H) have been identified in pigs, with species A rotavirus (RVA) having the highest prevalence [3]. Porcine rotavirus A (PoRVA) infections are detected in herds across all ages and primarily cause severe diarrhea in weaning and suckling piglets [4]. PoRVA exhibits substantial clinical and pathological similarities to transmissible gastroenteritis virus, porcine epidemic diarrhea virus, and porcine delta coronavirus, complicating their differentiation [5,6]. Co-infections or secondary infections involving these viruses are prevalent, cause economic losses to the livestock industry, and threaten human and animal health [7].

PoRVA remains prevalent in China and lives in pigs without causing clinical signs of diarrhea [8]. Recent analyses of the predominant PoRVA genotypes in China revealed that the primary VP7 genotypes in Shandong Province were G3, G5, and G9 [9]. In Taiwan, the prevalent VP7 genotypes are G9, G3, G4, and G5, while P[13], P[19], and P[23] are the dominant VP4 types [10]. In East China, G9, G1, and G5 are the main VP7 genotypes, and P[7], P[13], and P[23] are the most common VP4 genotypes [11]. The G9 PoRVA genotype, now dominant in Chinese pig herds, raises concerns about potential cross-species transmission between humans and pigs [12,13,14,15]. However, a vaccine targeting the G9-type PoRVA is currently unavailable in China.

Vaccination remains the primary strategy for preventing RV infections [16]. Compared to oral live-attenuated vaccines, inactivated rotavirus vaccines (IRV) are safer as they eliminate contamination risks and prevent the virus from regaining virulence [17]. They also avoid severe adverse reactions like intussusception [18,19,20]. Previous studies have demonstrated that IRV immunization induces the production of neutralizing anti-RV antibodies in mice and rhesus monkeys [21,22]. Additionally, IRV formulation for parenteral administration has received significant attention [23]. A vaccine candidate based on the G1P[8] strain has been tested in several animal models involving mice, rats, rabbits, and pigs, where it successfully triggered neutralizing antibody responses against various RV genotypes [24]. IRV immunization induces neutralizing antibodies in mice and rhesus monkeys, while a G1P[8]-based candidate vaccine has shown broad-spectrum cross-protection in multiple animal models. Current research on inactivated PoRVA vaccines, especially for emerging G9 strains, still has major knowledge gaps [25]. Key challenges like optimal inactivant selection, immunization protocol establishment, and sustained protective effects remain unresolved.

This study isolated and characterized the G9P[23] PoRVA strain RHeN2 from diarrheic piglets in a Henan swine farm, systematically evaluating its immunogenicity. Candidate vaccines were prepared using formaldehyde and binary ethylenimine (BEI) as inactivants. Neutralizing antibody levels in immunized piglets were dynamically monitored, and cross-neutralization assays against 11 heterologous PoRVA strains were conducted using sera collected at 31 days post-immunization to assess cross-protective capacity. These findings provide critical data for optimizing PoRVA-inactivated vaccine production processes and establish a scientific foundation for developing G9 genotype-specific vaccines.

## 2. Materials and Methods

### 2.1. Cells, Antibodies, and Viruses

The MA-104 cells used for isolating and propagating PoRVA were grown in a Dulbecco’s modified eagle medium (DMEM; Gibco, Grand Island, NY, USA), supplemented with 10% fetal calf serum (OPCEL, Hohhot, China). The anti-PoRVA VP6 protein monoclonal antibody (VP6-3B8) preserved in our laboratory, as well as a monoclonal antibody (R-4C2) utilizing different PoRVA genotypes provided by Dr. Wen Huang from Huazhong Agricultural University, were prepared as described here. First, BALB/c mice were immunized with PoRVA VP6 protein or inactivated PoRVA viral particles. Subsequently, their splenocytes were fused with Sp2/0 myeloma cells to generate hybridoma cells, which were screened and selected. The PoRVA strains RShanD1, ShanX3, RFuJ4, RHuN6, RHaiN7, and RHeN8 were isolated from clinical piglet diarrhea samples and stored in our laboratory. Strains KQ1 (G1P[7]), KQ2 (G3P[23]), KQ3 (G4P[23]), KQ4 (G5P[23]), and KQ5 (G9P[23]) were kindly provided by Wuhan Keqian Biology Co., Ltd. (Wuhan, China).

### 2.2. Clinical Samples Preparation

The PoRVA strain was isolated from five intestinal tissue samples of piglets with diarrhea, which were obtained from large-scale pig farms in Henan Province, China, in late 2021. Viral RNA was extracted using the TRIzol reagent, and cDNA was subsequently synthesized with the TaKaRa RNA PCR Kit (AMV) (Ver.3.0, TaKaRa Biomedical Technology, Beijing, China), following the manufacturer’s guidelines. The presence of viral nucleic acids was confirmed through RT-PCR, using the synthesized cDNA as templates and primers targeting the PoRVA VP7 gene (Table 1) [26]. For viral isolation, the inoculum was prepared by mixing 5 mL of DMEM with 1 mL of intestinal contents positive for PoRVA. The mixture was vortexed for 5 min, centrifuged at 4000× *g* and 4 °C for 10 min to remove cellular debris, and filtered through a 0.22-μm filter. The resulting filtered supernatant was used as the inoculum for PoRVA isolation.

### 2.3. Virus Isolation

PoRVA isolation was performed using MA-104 cells. The filtered supernatant (500 µL) was combined with trypsin (10 µg/mL), vortexed to ensure thorough mixing, and incubated for 1 h at 37 °C in a 5% CO_2_ atmosphere [27]. MA-104 cells cultured in six-well plates were washed with phosphate-buffered saline (PBS) and inoculated with the prepared supernatant. Each well was supplemented with 1.5 mL of a serum-free DMEM containing 3.33 µg/mL trypsin. After 1 h of virus adsorption, the cells were washed twice with PBS to remove unbound viral particles, followed by the addition of 2 mL of a serum-free DMEM containing 5 µg/mL trypsin. Cells were monitored daily for cytopathic effects (CPEs) until harvest.

After harvesting, the cell culture was subjected to three freeze-thaw cycles to facilitate viral release. Subsequently, the sample was centrifuged at 5000× *g* for 10 min, and the supernatant was collected. This supernatant was aliquoted into smaller volumes and stored at −80 °C to serve as the F0 generation virus. Serial passaging was performed in T25 cell culture flasks for virus propagation. If CPEs were not observed within 4 days post-inoculation, the flasks underwent three additional freeze-thaw cycles, followed by centrifugation and supernatant collection, enabling continued passaging for three blind passages, after which a PCR assay was performed. If the assay yielded positive results, passaging was continued; negative results led to the discarding of the inoculated cells.

### 2.4. Immunofluorescence Assay (IFA)

PoRVA isolates were inoculated into MA-104 cells in 24-well plates at a multiplicity of infection (MOI) of 0.1, with a negative control established after 24 h. The cells were fixed with 4% paraformaldehyde, permeabilized with methanol, and blocked with 5% Bovine serum albumin (BSA). After three washes with PBS, the cells were incubated with a monoclonal antibody (dilution: 1000×) and a FITC-conjugated secondary antibody. The nuclei were counterstained with DAPI, and fluorescence images were captured using a fluorescence microscope (Nikon, Tokyo, Japan).

### 2.5. Transmission Electron Microscopic (TEM) Observation

The virus-containing supernatant was centrifuged at 3000× *g* and 4 °C for 10 min, followed by filtration through a 0.22-µm membrane to remove cell debris. The filtered supernatant was added to an ultrafiltration device (Millipore, Amicon Ultra, Boston, MA, USA) and centrifuged at 4000× *g* and 4 °C for 10 min (repeated as necessary) until the sample volume was reduced to the target volume. Subsequently, the ultrafiltration device was inverted and placed into a new collection tube, followed by centrifugation at 1000× *g* for 2 min to collect the concentrated virus sample. Next, the concentrated sample was subjected to initial purification using a Capto Core 700 chromatography column (Cytiva, Marlborough, MA, USA), followed by further purification using an AKTA protein purification system. After purification, the virus concentration was measured using a NanoDrop 2000 spectrophotometer (Thermo Scientific^TM^, Waltham, MA, USA). To observe the virus morphology, 10 µL of the purified virus suspension was carefully applied to a copper grid and incubated for 10 min. Furthermore, 10 µL of phosphotungstic acid solution was added for negative staining, followed by an additional 10-min incubation. Finally, the virus samples were observed using a Hitachi H-7650 transmission electron microscope (Tokyo, Japan).

### 2.6. Viral Growth Curve Determination

The 10th passage of the PoRVA isolate was inoculated onto MA-104 cells in 24-well plates at an MOI of 0.2. Supernatants were harvested at various time points (8, 16, 24, 28, 32, 36, 40, 44, 52, and 60 h post-inoculation, hpi) to determine the median tissue culture infective dose (TCID_50_). The procedure was as follows: First, the cell monolayer was washed three times with PBS. Then, 100 µL of the supernatants was mixed with 900 µL of DMEM to prepare serial dilutions from 10^−1^ to 10^−9^. Each dilution was inoculated onto the cell monolayer at 100 µL per well, with eight replicates per dilution, alongside negative controls containing only medium. The plate was incubated at 37 °C, and CPEs were observed and recorded daily under a microscope for 5 days to determine the viral titers. Each time point was independently measured in triplicate. Finally, TCID_50_ was calculated using the Reed–Muench method [28]. The growth curve of the isolate was plotted using GraphPad Prism 8.4.3 (GraphPad Software, La Jolla, CA, USA).

### 2.7. Whole-Genome Sequencing Analysis

The total RNA was extracted from the cell culture samples using Omega Bio-tek^®^ Total RNA Extraction Kit I (Omega Biotech, Norcross, GA, USA). Reverse transcription to cDNA was performed with Reverse Transcription Kit R212 (Vazyme, Nanjing, China). Based on the conserved regions of the entire PoRVA genome, 11 pairs of specific primers were designed (Table 2). High-fidelity amplification was performed using Phanta Max^®^ Super-Fidelity DNA Polymerase P505 (Vazyme Biotech Co., Ltd., Nanjing, China). The amplified bands were purified using EasyPure^®^ PCR Purification Kit (TransGen Biotech Co., Ltd., Beijing, China). The purified products were sent to Qingke Biotechnology Co., Ltd. (Beijing, China) for Sanger sequencing. After the raw data were assembled by SeqMan (DNASTAR Lasergene 17, Madison, WI, USA), multiple sequence alignment and phylogenetic analysis were conducted using MEGA11 [29].

### 2.8. Neutralization Test

The virus neutralization assay was conducted in 96-well cell culture plates to determine the serum-neutralizing antibody titers. MA-104 cells were seeded at a density of 2 × 10^4^ cells/well and cultured for 24 h. The RHeN2 strain culture was diluted to 200 TCID_50_/50 µL using serum-free DMEM. Equal volumes (50 µL) of the diluted virus and serially diluted serum samples (starting from 1:8 to 1:2048) were mixed in a 96-well plate and incubated at 37 °C for 1 h. The virus–serum mixture was then added to the MA-104 cell monolayer and incubated at 37 °C for 1 h. After incubation, the cells were washed three times with PBS and maintained in DMEM supplemented with 2% fetal bovine serum at 37 °C in a 5% CO_2_ incubator for 72 h. The neutralizing antibody titer (ND_50_) was determined using the Reed–Muench method, defined as the highest serum dilution that inhibited 50% of the CPEs [28].

### 2.9. Preparation of Inactivated Vaccines

For the BEI-inactivated vaccine, bromoethylamine hydrobromide was first mixed with a PBS solution containing 0.5 M sodium hydroxide and incubated for 1 h in a 37 °C water bath to generate a 0.2 M BEI inactivating agent, which was filtered and sterilized for later use. Subsequently, the BEI inactivating agent was added to the purified RHeN2 virus solution at a volume ratio of 1:40, followed by inactivation for 48 h at 37 °C. The reaction was terminated by adding sodium thiosulfate to a final concentration of 2% [30]. For the formaldehyde-inactivated vaccine, the purified virus solution was mixed with 0.2% formaldehyde and inactivated for 72 h at 37 °C. Next, 1% sodium bisulfite was slowly added, and the mixture was allowed to react for 30 min at 37 °C to completely neutralize the formaldehyde, forming non-toxic hydroxymethyl sulfonate. The neutralization effect was verified using a potassium iodide-starch test paper. The inactivated virus solution was inoculated onto MA-104 cells and observed for 4–6 days. If no CPE was observed, the solution was blindly passaged for three additional generations to confirm the absence of infectivity, while sterility testing was conducted to ensure aseptic conditions. Finally, an equal proportion of adjuvant (Montanide™ ISA 201^®^, SEPPIC, Paris, France) was added to both inactivated vaccines for formulation. The entire preparation process strictly followed inactivation, neutralization, and safety verification steps to ensure complete virus inactivation, thorough removal of formaldehyde residues, and validation of the vaccine’s safety and immunogenicity.

### 2.10. Immunization of Piglets with Inactivated PoRVA RHeN2 Vaccine

Fifteen 28-day-old piglets with PoRVA-neutralizing antibody titers ≤1:10 were randomly assigned to three groups (Table 3). A randomized controlled trial was conducted to assess the immunogenicity of the PoRVA-inactivated vaccine. The experimental group (*n* = 10) was intramuscularly injected with the inactivated vaccine containing 50% adjuvant (2 mL/dose), followed by a second immunization using the same method and dose after 2 weeks. The control group (*n* = 5) received an intramuscular injection of DMEM containing 50% adjuvant (2 mL/dose). Serum was collected at 15 weeks to assess the immune response. As described in Section 2.8, serum neutralization titers were determined for each sample using RHeN2 strain.

### 2.11. Statistical Analysis

Statistical analyses were performed using GraphPad Prism 8.4.3 (GraphPad Software, CA, USA). Multiple comparisons were conducted using Student’s *t*-test and one-way/two-way analysis of variance. The data were presented as means ± standard deviations, and *p* < 0.05 was considered statistically significant.

## 3. Results

### 3.1. Virus Isolation

**Figure 1:**
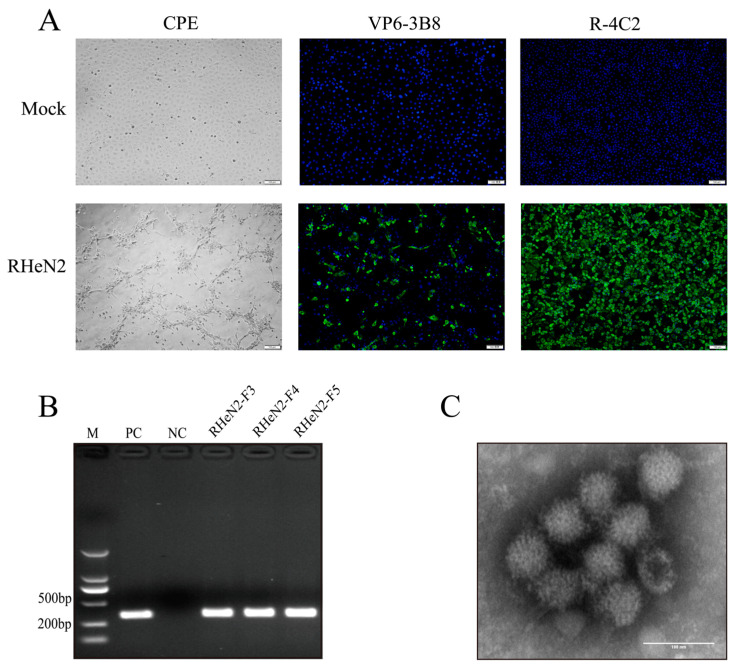
Isolation and characterization of porcine rotavirus RHeN2 from piglets exhibiting clinical diarrhea. (**A**) Cytopathic effects induced by RHeN2 infection in MA-104 cells and immunofluorescence detection (Monoclonal antibodies: VP6-3B8 specific to PoRVA VP6 protein; R-4C2 specific to inactivated PoRVA virions) Scale bar: 100 µm (**B**) Detection of RHeN2 in viral supernatants from F3–F5 passages following blind transmission using RT-PCR with VP7-specific primers. (**C**) Transmission electron micrograph of the purified RHeN2 strain, revealing the characteristic viral morphology. Scale bar: 100 nm. Figures S1–S3.

The virus was isolated from five PoRVA samples that were RT-PCR positive. In the blind passages of the third generation of MA-104 cells, samples from Henan showed CPEs primarily characterized by cell rounding, aggregation, lysis, and detachment (Figure 1A), consistent with the CPEs caused by PoRVA. It was named the PoRVA RHeN2 strain based on its origin. RT-PCR detection of the F3–F5 generations of the viral solution from the strains produced specific amplification bands of 333 bp (Figure 1B), indicating that the isolated virus was PoRVA and could proliferate stably in MA-104 cells.

The proliferation of the PoRVA virulent strain RHeN2 in MA-104 cells was confirmed through IFA using a monoclonal antibody targeting the PoRVA VP6 protein (Figure 1A). Immunofluorescence specific for the PoRVA VP6 protein was observed in most cells 16 hpi, with the VP6 protein predominantly localized in the cytoplasm. In contrast, no fluorescence was detected in the control group.

TEM revealed particles of the purified RHeN2 virus strain infecting MA-104 cells. Negative staining under electron microscopy revealed typical wheel-shaped particles with a radial arrangement from the center to the periphery, measuring approximately 70 nm in diameter (Figure 1C).

### 3.2. Virus Growth Characterization

**Figure 2:**
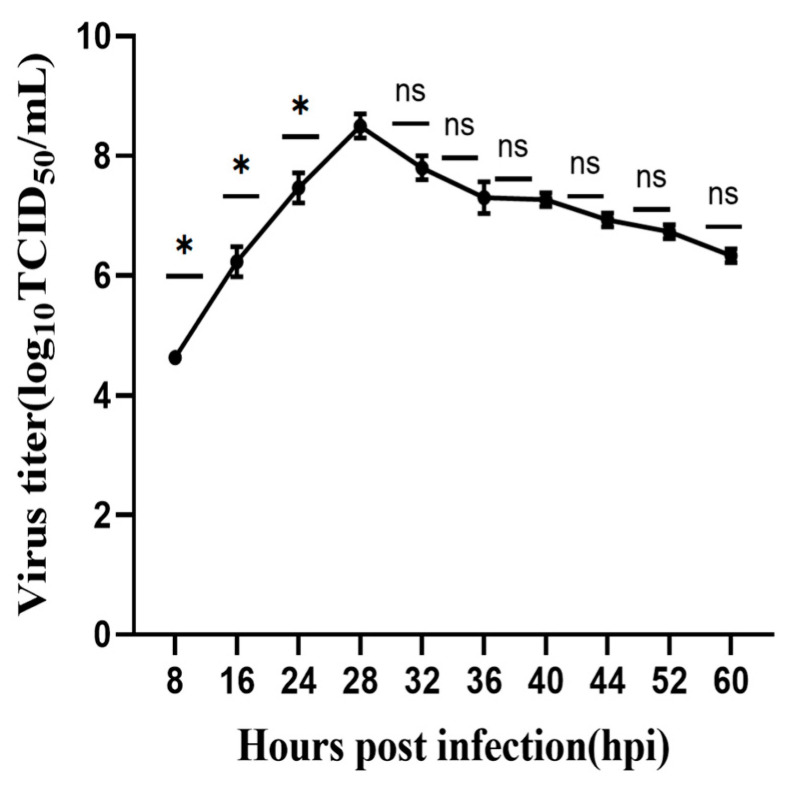
Growth kinetics of RHeN2 in MA-104 cells (MOI = 0.2). Data represent the mean of three independent experiments, with error bars representing standard deviation. ns: not significant; * *p* < 0.05.

To elucidate the growth kinetics of the RHeN2 strain, MA-104 cells were infected with the 10th passage of RHeN2 at an MOI of 0.2, and viral titers were measured at various time points to generate a growth curve (Figure 2). The results revealed a typical biphasic replication pattern: during the 8–28 hpi period, the average viral titer increased significantly from 10^4.63^ TCID_50_/mL to a peak of 10^8.5^ TCID_50_/mL (*p* < 0.05), indicating rapid viral replication during the early infection phase. After 28 hpi, the virus entered a plateau phase, with no significant differences in viral titers between adjacent time points (*p* > 0.05), suggesting a dynamic equilibrium between viral replication and host cell antiviral responses. Notably, this replication pattern remained stable during subsequent passages, with the peak titer consistently observed at 28 hpi, confirming that the RHeN2 strain exhibits stable growth kinetics.

### 3.3. Whole-Genome Sequence of the RHeN2 Strain

The isolate was confirmed as PoRVA through Sanger sequencing and subsequent BLAST analysis against GenBank entries (Table 4). The genomic sequence of this strain (G9-P[23]-I5-R1-C1-M1-A8-N1-T1-E1-H1) has been deposited in GenBank under accession numbers PV026137 to PV026147. Comparative analysis of coding sequences revealed distinct phylogenetic relationships: eight gene segments (VP1, VP2, VP4, VP6, VP7, NSP2, NSP4, and NSP5) exhibited the highest nucleotide identity to PoRVA reference strains (99.94%, 97.23%, 95.71%, 96.21%, 99.68%, 97.36%, 97.97%, and 97.74%, respectively). In contrast, three segments (VP3, NSP1, and NSP3) showed closer homology to human rotavirus A (HRVA) strains, with nucleotide identities of 96.84%, 95.57%, and 96.83%, respectively.

### 3.4. Phylogenetic Analysis

**Figure 3:**
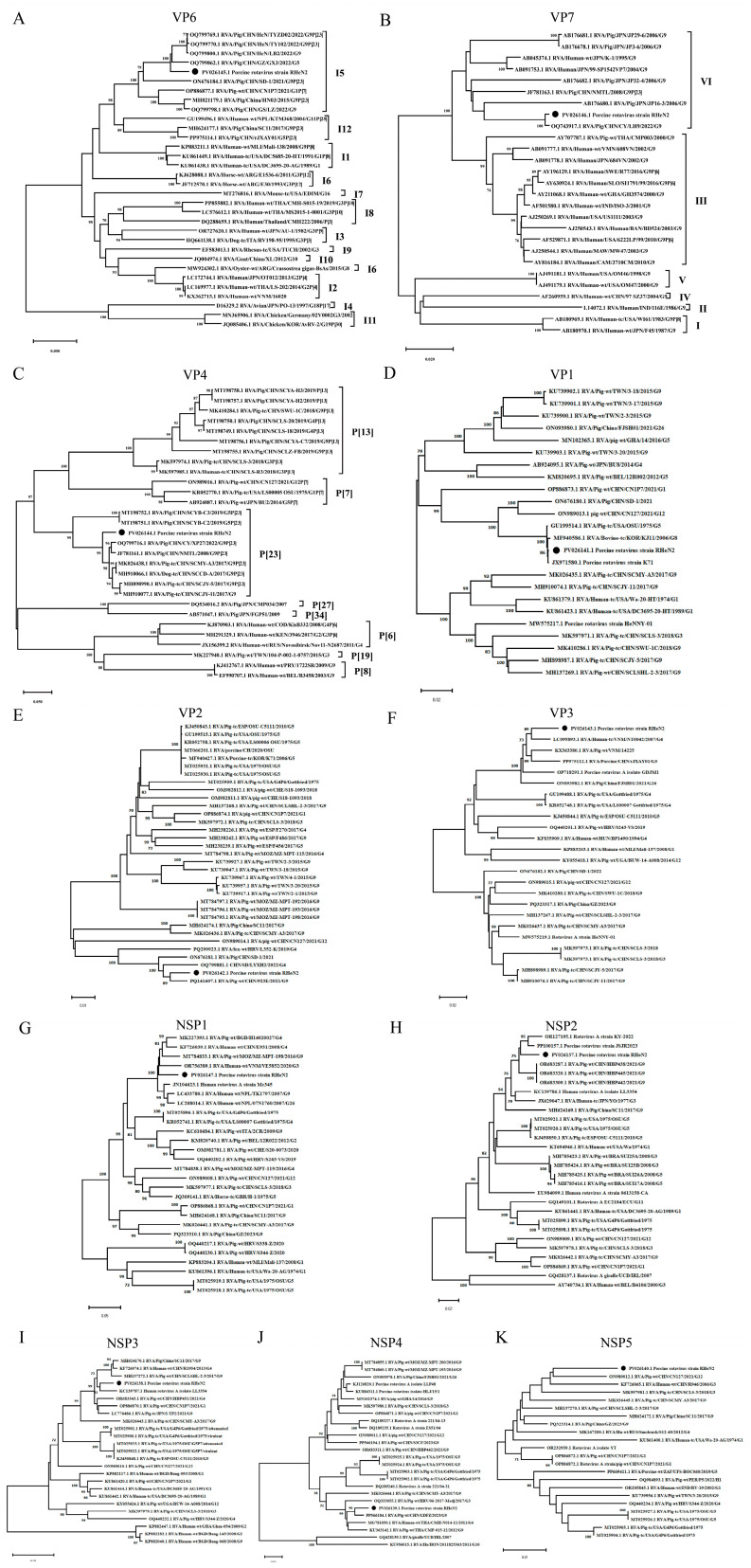
Phylogenetic analysis of RHeN2 and other representative strains based on the nucleotide sequences of 11 genome segments, performed using MEGA11 and the neighbor-joining (NJ) method. (**A**–**K**) Phylogenic trees based on the nucleotide of VP6, VP7, VP4, VP1, VP2, VP3, NSP1, NSP2, NSP3, NSP4 and NSP5 genes from RHeN2.

The RHeN2 strain was classified as I5 via the analysis of the VP6 gene, demonstrating the highest nucleotide homology (96.21% nt) with the porcine RV CHN/GZ/GX3/2022/G5 (Figure 3A). Regarding the VP7 gene, there are six recognized VP7 G9 lineages (Lineages I–VI) [31]. RHeN2 was placed within Lineage VI of the six recognized VP7 G9 lineages (Figure 3B), showing close alignment with the Chinese strain CHN/CY/LH9/2022/G9, with a nucleotide identity of 99.68%. For the VP4 gene, RHeN2 exhibited a nucleotide identity of 95.71% with the Chinese strain RVA/Pig/China/NMTL/2008/G9P[23] (Figure 3C). Concerning VP1, RHeN2 shared the highest nucleotide identity (99.94% nt) with the porcine strain K71 (Figure 3D). The VP2 gene of RHeN2 exhibited close clustering with the porcine strain RVA/Pig-wt/CHN/923E/2021/G9P[23], demonstrating a nucleotide identity of 97.23% (Figure 3E). For the VP3 gene, RHeN2 was most similar to the human strain RVA/human-tc/VNM/NT0042/2007/G4P[6], with a nucleotide identity of 96.84% (Figure 3F). Considering NSP1, RHeN2 displayed the highest homology (95.57% nt) with the human-origin strain Mc345 (Figure 3G). The NSP2 gene of RHeN2 exhibited the highest similarity degree to the porcine strain JSJR2023, sharing a nucleotide identity of 97.36% (Figure 3H). For NSP3, RHeN2 clustered closely with the human strain RVA LL3345, with a nucleotide identity of 96.83% (Figure 3I). Analysis of NSP4 revealed that RHeN2 exhibited the highest homology (97.97% nt) with the porcine strain RVA/Pig-wt/CHN/GDFZ/2023/G9P[23] (Figure 3J). Finally, for the NSP5 gene, RHeN2 showed the greatest similarity (97.74% nt) with the Chinese strain RVA/Pig-wt/CHN/CN127/2021/G12P[7] (Figure 3K).

### 3.5. Immunogenicity of the RHeN2 Strain in Piglets

**Figure 4:**
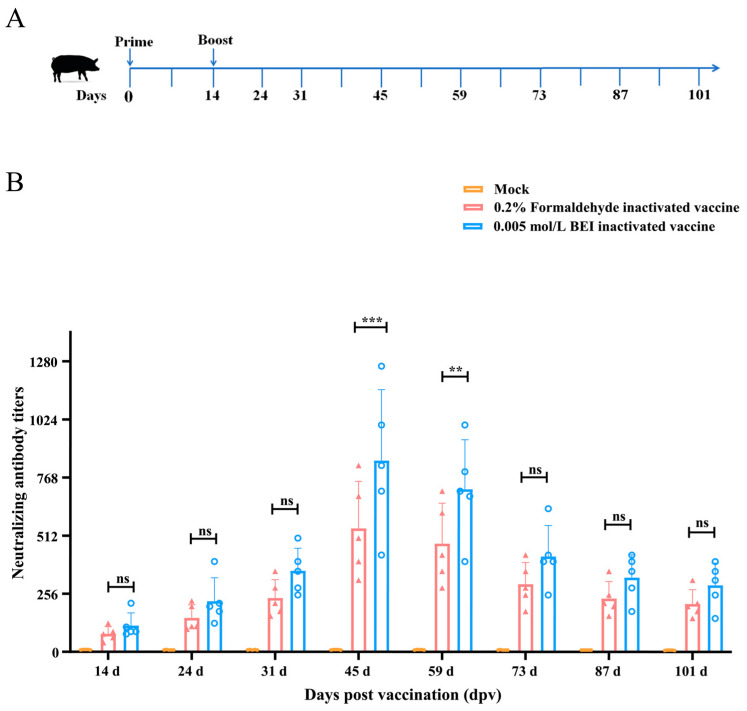
Evaluation of the immunogenicity of the PoRVA RHeN2 strain. (**A**) Schematic representation of the animal experiment, including the immunization protocol and sample collection timeline. (**B**) Measurement of specific neutralizing antibody levels against the RHeN2 strain in the serum of piglets vaccinated with the inactivated PoRVA vaccine at various time points. ns: not significant; ** *p* < 0.01, *** *p* < 0.001.

To assess the immunogenicity of the RHeN2 strain and the effects of various inactivators on its immunological potential, animal trials were conducted according to the methodology outlined in the Materials and Methods section. Piglets (28 days old) were intramuscularly administered with 2 mL of RHeN2 that had been inactivated using 0.005 mol/L BEI, 0.2% formaldehyde, or DMEM as the control. A booster dose was administered 2 weeks after the initial injection. Serum samples were collected on designated days post-vaccination (dpv), and serum neutralization titers were measured (Figure 4A). At 14 dpv, neutralizing antibodies, with titers no less than 1:40, were detected in the immunized piglets (Figure 4B). Antibody levels continued to increase and peaked at 45 dpv. The mean neutralizing antibody titers in the BEI-inactivated vaccine group were significantly greater than those in the formaldehyde-inactivated vaccine group. The BEI-inactivated group had an average neutralizing titer of 1:843, while the formaldehyde-inactivated group showed a titer of 1:544.4. By 59 dpv, serum-neutralizing antibody levels began to decline gradually. At 73 dpv, a more significant decrease was observed, although antibody levels remained relatively high through 101 dpv. At this point, the mean neutralizing antibody titer in the BEI-inactivated vaccine group was 1:299.6, whereas the formaldehyde-inactivated vaccine group had a titer of 1:211.2. The results demonstrate that the inactivated vaccine prepared from the PoRVA RHeN2 strain exhibits favorable immunogenicity. As a vaccine inactivator, BEI shows superior efficacy compared to the traditional formaldehyde inactivation method. BEI more effectively preserves the immunogenic epitopes of the virus, thereby inducing a relatively stronger and more sustained immune response in the host.

### 3.6. The Cross-Neutralizing Activity of the Inactivated RHeN2 Vaccine Against 11 Strains of PoRVA Strains

**Figure 5:**
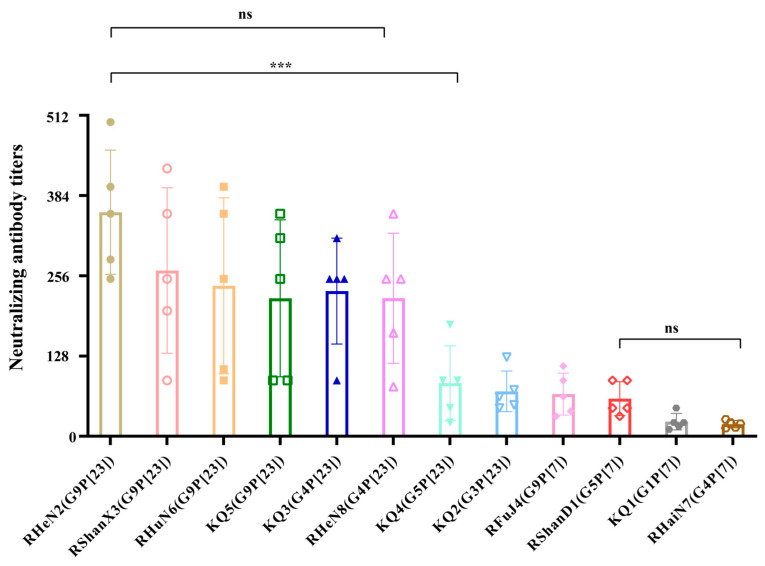
Neutralizing antibody titers of the PoRVA RHeN2 strain inactivated vaccine against 11 PoRVA strains. Data represent the neutralizing antibody titers against different PoRVA strains in serum from 5 piglets immunized with BEI-inactivated vaccine at 31 days post-vaccination. Error bars indicate standard deviation. ns: not significant; *** *p* < 0.001.

To evaluate the cross-protective efficacy of the PoRVA RHeN2 (G9P[23]) inactivated vaccine against other PoRVA strains, we conducted a cross-neutralization assay using sera collected from five piglets in the BEI-inactivated vaccine group (31 days post-vaccination) against 11 different PoRVA strains [RShanD1 (G5P[7]), RShanX3 (G9P[23]), RFuJ4 (G9P[7]), RHuN6 (G9P[23]), RHaiN7 (G4P[7]), RHeN8 (G4P[23]), KQ1 (G1P[7]), KQ2 (G3P[23]), KQ3 (G4P[23]), KQ4 (G5P[23]), and KQ5 (G9P[23])] (Figure 5). Analysis of neutralizing antibody levels revealed that the immune sera exhibited the highest neutralizing activity against the homologous strain RHeN2 (G9P[23]), with an average titer of 357.4. The sera also showed high neutralizing activity against strains of the same genotype (G9P[23]), including RShanX3, RHuN6, and KQ5, with average titers of 264.4, 240.0, and 220.0, respectively, showing no significant difference compared to the homologous strain. Additionally, the immune sera demonstrated high neutralizing activity against G4P[23] genotype strains (RHeN8 and KQ3), with average titers of 220.2 and 231.6, respectively, and no significant difference compared to the homologous strain. However, the neutralizing activity against strains of other genotypes was significantly reduced (*p* < 0.0001), with average titers of 84.6, 71.6, 67.2, 60.0, 23.2, and 19.2 against G5P[23] (KQ4), G3P[23] (KQ2), G9P[7] (RFuJ4), G5P[7] (RShanD1), G1P[7] (KQ1), and G4P[7] (RHaiN7) strains, respectively. These results indicate that the RHeN2 (G9P[23]) inactivated vaccine provides significant cross-protection against G9P[23] and G4P[23] genotype strains but offers limited protection against strains of other genotypes. This finding provides important insights for the clinical application and optimization of immunization strategies for PoRVA G9-type vaccines.

## 4. Discussion

The isolation and propagation of G9-type in cell cultures are usually challenging, possibly owing to its high dependence on trypsin [32]. Trypsin plays a critical role in RV infection by cleaving the viral surface protein VP4 into VP5* and VP8* polypeptides, enhancing virus–host cell interaction and infectivity [33]. Direct virus inoculation onto cells results in low isolation efficiency. To address this, we introduced a trypsin pre-treatment approach and optimized trypsin concentration, significantly improving PoRVA isolation and propagation in MA-104 cells. Experiments showed stable virus propagation at trypsin concentrations ≥5 µg/mL. However, to mitigate potential cytotoxicity (e.g., altered cell morphology and reduced viability), we used 10 µg/mL trypsin during pre-treatment and reduced it to 5 µg/mL during cell inoculation. This optimized protocol provides reliable technical support for further research and represents a valuable reference for isolating other challenging RV strains.

The detection rate of PoRVA in pig farms has increased recently, and the prevalent genotypes have changed significantly, evolving from a predominantly G5-type to a predominantly G9-type [34,35]. In this study, PoRVA strains were isolated from the intestinal samples of piglets with diarrhea in Henan Province, China. We sequenced the VP4 and VP7 genes of these strains and identified them as the G9P[23] type. The emergence of new strains poses an economic threat to the livestock industry and a risk to human and animal health. Studies have demonstrated that G9 PoRVA offers complete short-term cross-protection against HRV and PoRVA, while HRV provides only partial protection against PoRVA [36]. Therefore, developing a vaccine specifically aimed at G9 PoRVA is vital for effectively managing its spread. Furthermore, genomic analyses reveal that G9 strains exhibit dual tropism, infecting both pigs and humans, with significant genetic diversity observed among circulating strains [37,38]. The presence of recombinant variants in pig populations and human cohorts highlights the potential for cross-species transmission, driven by genetic similarities between pig and human G9 strains. Notably, we observed that the VP3, NSP1, and NSP3 proteins of the RHeN2 strain are highly homologous with those of HRVA. Moreover, phylogenetic analyses of human G9 RVs have shown that they are more closely related to porcine G9 strains than to earlier human G9-types [39]. These findings underscore the potential public health risks posed by PoRVA.

Vaccination remains the primary strategy for preventing severe diarrhea caused by RV. Inactivated vaccines offer several advantages over live vaccines, including a reduced risk of contamination by exogenous factors and the absence of concerns related to virulence reversion. Notably, inactivated vaccines effectively mitigate the occurrence of severe adverse reactions, such as intussusception, and can be easily combined with other vaccines. These characteristics are crucial for the effective control of RV infections and the reduction of mortality rates associated with severe diarrhea in piglets. To evaluate the immunogenicity of the inactivated vaccine of the virulent strain, we used two inactivation methods to select the inactivating agent: 2% formaldehyde and 5 mM BEI. By comparing the inactivation effects of formaldehyde and BEI on PoRVA, we provided a valuable reference for research on the inactivation process for the development of a PoRVA-inactivated vaccine. Formaldehyde is the most common inactivation agent [40]. BEI interacts with nucleic acids, destroying their structure and completely inactivating the virus. Meanwhile, it does not damage the surface structural proteins of the virus, which retain their antigenicity, and BEI does not remain in the host [41]. Throughout the detection period, neutralizing antibody levels in the BEI-inactivated vaccine group were consistently higher than those in the formaldehyde-inactivated vaccine group. This suggests that BEI inactivation had a lesser effect on the immunogenicity of the PoRVA G9P[23] strain than formaldehyde inactivation. These findings offer valuable insights for selecting appropriate inactivating agents in the development of future inactivated vaccines targeting the G9P[23] strain. We tested the cross-neutralizing activity of the inactivated G9P[23] RV vaccine with prevalent strains of multiple other genotypes in piglets. The results showed that the inactivated RHeN2 vaccine had a strong cross-neutralizing ability against the same G9P[23] and G4P[23] strains and a stronger cross-neutralizing ability against the P[23] strains than against the P[7] strains. This suggests that the RHeN2 (G9P[23]) immune serum produces a good neutralizing response against the currently prevalent strains of multiple genotypes and represents a promising candidate vaccine strain.

## 5. Conclusions

The PoRVA strain RHeN2 (G9P[23]) was successfully isolated and subcultured in MA-104 cells. Its biological characteristics were characterized through IFA and TEM, with the viral titer peaking at 28 hpi. Whole-genome sequencing revealed the RHeN2 strain as G9-P[23]-I5-R1-C1-M1-A8-N1-T1-E1-H1. Animal experiments demonstrated that the PoRVA RHeN2 strain exhibits strong immunogenicity and significant cross-neutralizing activity against other strains of the same genotype and G4P[23] strains. These findings lay the foundation for future research on vaccine development, G9-type PoRVA, and its pathogenic mechanisms.

## Figures and Tables

**Table 1:** Primer sequences for the specific amplification of PoRVA.

Primer	Primer Sequences (5′→3′)	Product (bp)
PoRVA-VP7-F	CCCCGGTATTGAATATACCACAGT	333
PoRVA-VP7-R	TTTCTGTTGGCCACCCTTTAGT	

**Table 2:** Primer sequences used for the amplification of the full-length genome of PoRVA.

Primer	Primer Sequences (5′→3′)	Product (bp)
VP1-F	GGCTATTAAAGCTRTACAATGGGGAAG	3302
VP1-R	GGTCACATCTAAGCGCTCTAATCTTS	
VP2-F	GGCTATTRAAGGYTCAATGGCGTACAG	2690
VP2-R	GTCATATCTCCACARTGGGGTTGG	
VP3-F	GGCTWTTAAAGCARTATTAGTAGTG	2591
VP3-R	GGTCACATCATGACTAGTGTG	
VP4-F	GGCTATAAAATGGCTTCGCTAATTTACAG	2362
VP4-R	GGTCACATCCTTTAGAAGCTACTTATAGTCTACATTG	
VP6-F	GGCTTTWAAACGAAGTCTTC	1356
VP6-R	GGTCACATCCTCTCACT	
VP7-F	GGCTTTAAAAGAGAGAATTTC	1062
VP7-R	GGTCACATCATACAATTC	
NSP1-F	GGCTTTTTTTATGAAAAGTCTTGT	1581
NSP1-R	GGTTCACATTTTTTGCTACCTAGG	
NSP2-F	GGCTTTTAAAGCGTCTCAG	1059
NSP2-R	GGTCACATAAGCGCTTTC	
NSP3-F	GGCTTTTAATGCTTTTCAGTG	1104
NSP3-R	GGTCACATAACGCCCCTATAG	
NSP4-F	GGCTTTTAAAAGTTCTGTTCC	751
NSP4-R	GGWYACRYTAAGACCRTTCC	
NSP5-F	GGCTTTTAAAGCGCTACAG	667
NSP5-R	GGTCACAAAACGGGAGT	

**Table 3:** Design of the immunogenicity evaluation assay for the PoRVA RHeN2 strain.

Group	Number of Piglets	Immunization Dose
0.005mol/L BEI-inactivated	5	2 mL
0.2% formaldehyde-inactivated	5	
DMEM	5	

**Table 4:** Analysis of the individual fragments of the PoRVA RHeN2 strain.

Gene	Segment Number	Genotype	Best Hit Accession	Query Coverage%	Identity%
VP7	9	G9	CHN/CY/LH9/2022/G9 (OQ743917.1)	100	99.68
VP4	4	P[23]	RVA/Pig/China/NMTL/2008/G9P[23] (JF781161.1)	100	95.71
VP6	6	I5	CHN/GZ/GX3/2022/G5 (OQ799862.1)	100	96.21
VP1	1	R1	Porcine rotavirus strain K71 (JX971580.1)	100	99.94
VP2	2	C1	RVA/Pig-wt/CHN/923E/2021/G9P[23] (PQ141607.1)	99	97.23
VP3	3	M1	RVA/Human-tc/VNM/NT0042/2007/G4P[6] (LC095893.1)	100	96.84
NSP1	5	A8	Human rotavirus A strain Mc345 (JN104623.1)	100	95.57
NSP2	8	N1	Porcine rotavirus strain JSJR2023 (PP100157.1)	100	97.36
NSP3	7	T1	Human rotavirus A isolate LL3354 (KC139787.1)	99	96.83
NSP4	10	E1	RVA/Pig-wt/CHN/GDFZ/2023/G9P[23] (PP566184.1)	99	97.97
NSP5	11	H1	RVA/Pig-wt/CHN/CN127/2021/G12P[7] (ON989012.1)	100	97.74

## Data Availability

The data are contained within the article and Supplementary Materials.

## Supplementary Materials

The following supporting information can be downloaded at: https://www.mdpi.com/article/10.3390/vetsci12020180/s1, Figure S1: IFA-Mock; Figure S2: IFA-RHeN2; Figure S3: Original electron microscope image.
