# Supplementary figures and images for: Isolation, Genomic Characterization, and Immunogenicity Evaluation of a G9P[23] Porcine Rotavirus Strain
Source: Vet Sci. 2025 Feb 18;12(2):180. doi: 10.3390/vetsci12020180 (PMC11861734; doi:10.3390/vetsci12020180)

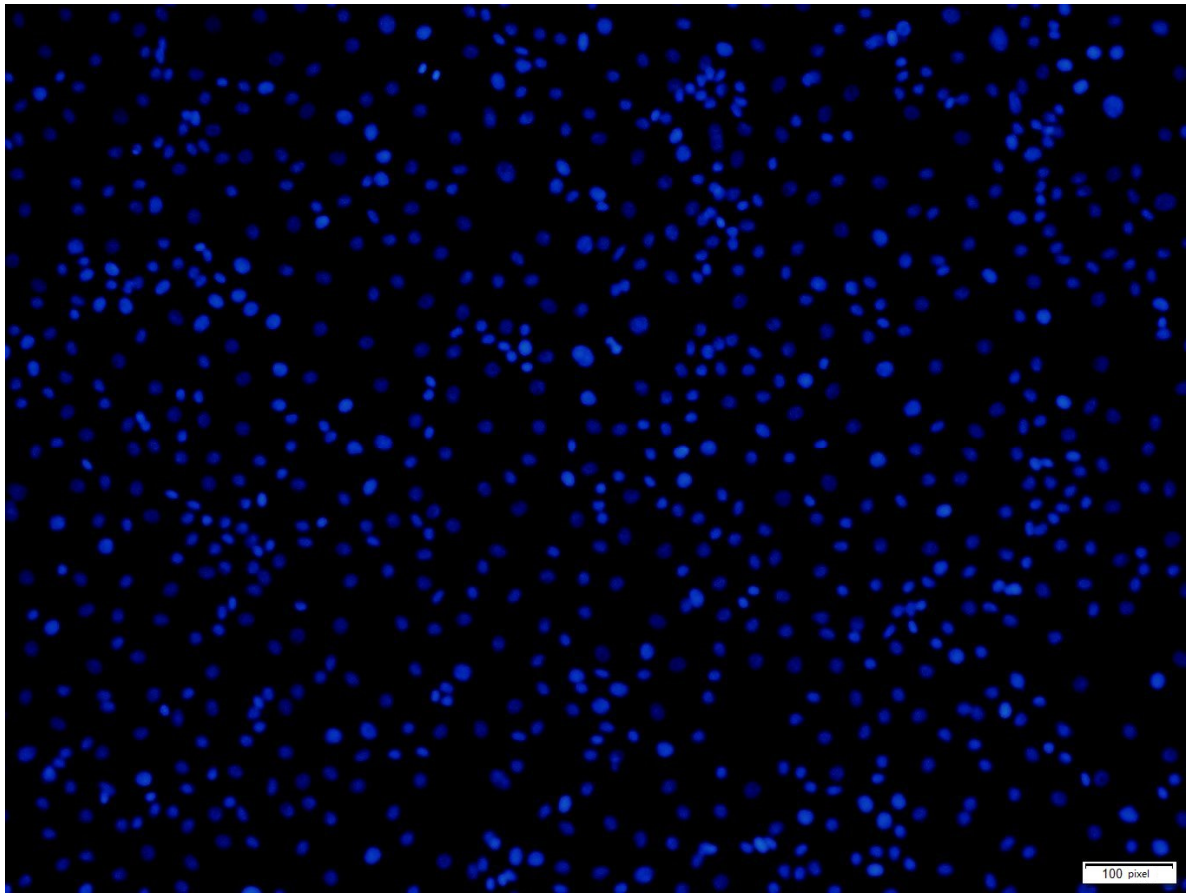

Figure S1: IFA-Mock.

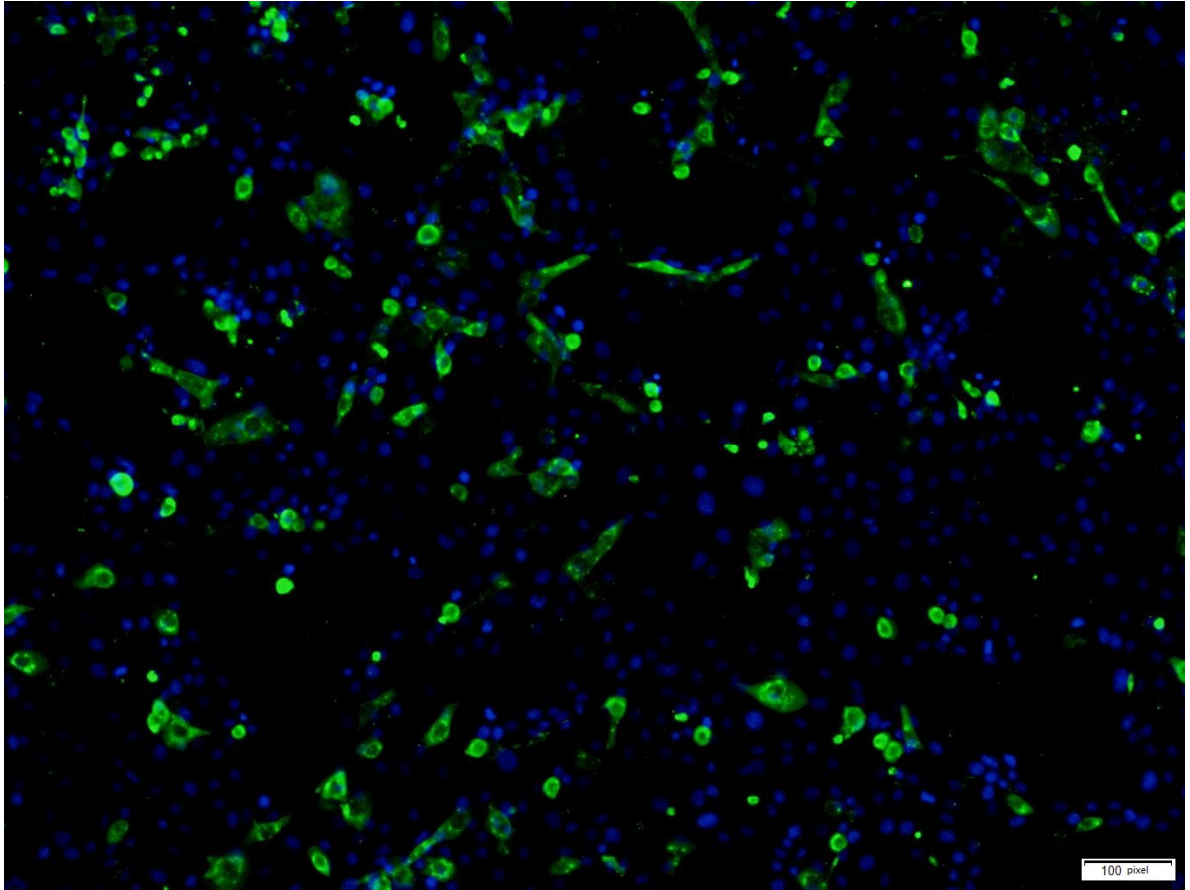

Figure S2: IFA-RHeN2.

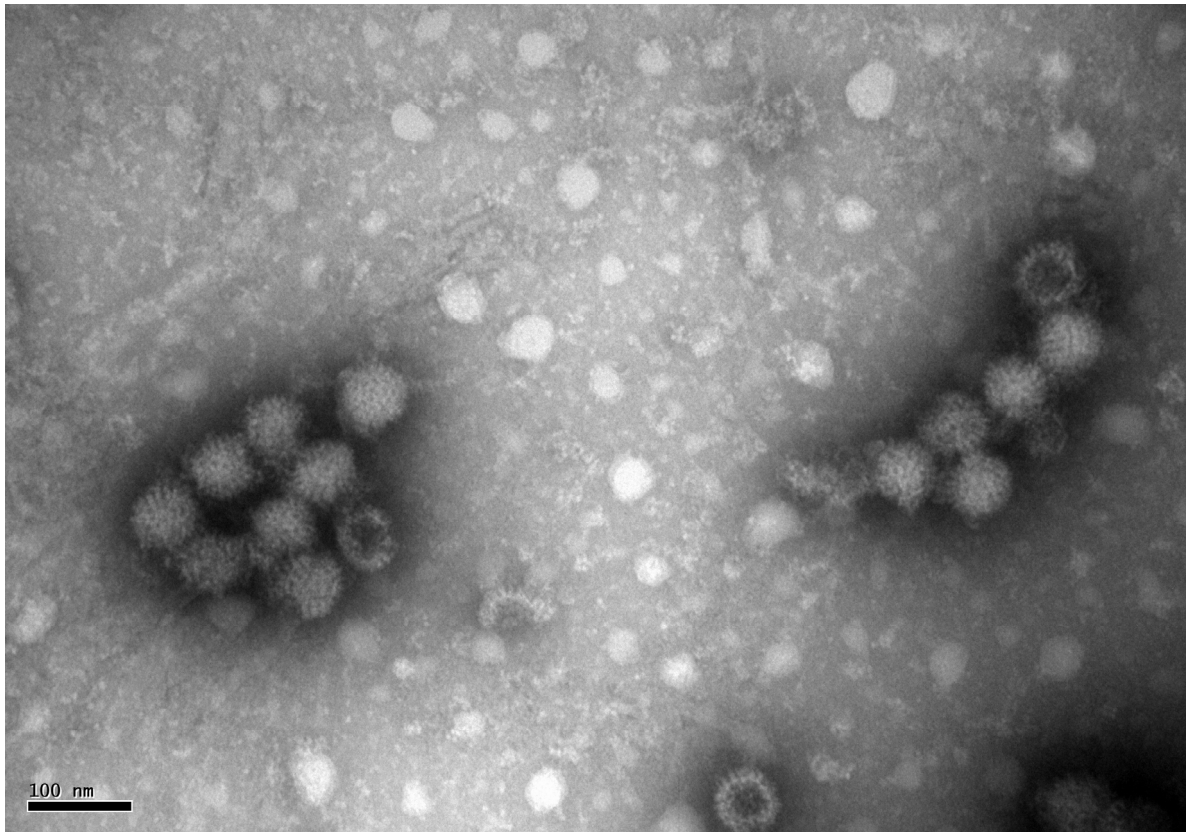

Figure S3: Original electron microscope image.

Supplement: Supplementary file 1 [file vetsci-12-00180-s001.zip › vetsci-3434010-supplementary.pdf]
